# Putative functional variants of lncRNA identified by RegulomeDB were associated with knee osteoarthritis susceptibility

**DOI:** 10.1186/s12891-018-2197-1

**Published:** 2018-08-07

**Authors:** Kejie Wang, Minjie Chu, Wenge Ding, Qing Jiang

**Affiliations:** 1 Department of Sports Medicine and Adult Reconstructive Surgery, Drum Tower Hospital Clinical College of Nanjing Medical University, Zhongshan Road 321, Nanjing, 210008 Jiangsu People’s Republic of China; 2 Department of Orthopaedics, Changzhou No.1 People’s Hospital, Changzhou, 213003 Jiangsu People’s Republic of China; 3 Department of Epidemiology, School of Public Health, Nantong University, Nantong, 226019 Jiangsu People’s Republic of China; 4 The Center of Diagnosis and Treatment for Joint Disease, Drum Tower Hospital Affiliated to Medical School of Nanjing University, Nanjing, 210008 Jiangsu People’s Republic of China

**Keywords:** lncRNA, Osteoarthritis, Polymorphism, H19, MEG3, HOTTIP

## Abstract

**Background:**

Knee osteoarthritis (KOA) is the most common form of chronic degenerative joint disease worldwide. Its incidence has increased in recent years. Aberrant expression profile of lncRNAs in damaged bone and cartilage of KOA patients has been reported recently, indicating its potential contributions in KOA development and a promising target for disease diagnosis and treatment. The aim of this study was to identify the association between genetic variation in lncRNA and KOA.

**Methods:**

We retrieved relevant articles from the PubMed, Medline and Embase databases up to Jul 2017 investigating the association between lncRNA and the risk of osteoarthritis. There are 15 lncRNAs which show connection with osteoarthritis. We selected potential functional polymorphisms identified by RegulomeDB database in these lncRNAs. A case-control study was conducted which contained 278 KOA patients and 289 OA-free controls.

**Results:**

Logistic regression analyses revealed that H19 rs2067051 T allele was significantly associated with decreased risk of KOA after adjusted for age, gender and BMI in recessive genetic model (OR = 0.63, *P* = 0.03) and additive genetic model (OR = 0.79, *P* = 0.03). MEG3 rs4378559 T allele was significantly associated with increased risk of KOA in additive genetic model (OR = 1.32, *P* = 0.04). Heterogeneity tests proved that H19 rs2067051, MEG3 rs4378559 and HOTTIP rs202384’s risk effects on KOA were more remarkable for female, BMI ≥ 25 and younger age (age < 60), respectively.

**Conclusion:**

The results indicate that potential functional genetic variation in lncRNA plays an important role in the pathogenesis of KOA.

## Background

Knee osteoarthritis (KOA) is the most common joint disease worldwide, characterized by progressive degeneration of articular cartilage, synovitis, osteophyte formation, and subchondral bone sclerosis [1]. It can cause severe pain and physical disability thus substantially reduce elder people’s quality of life [2, 3]. Although the incidence of osteoarthritis is still increasing, affecting approximately 10% of men and 18% of women over 60 years of age, its pathophysiology is still evolving and undetermined [4]. Twin-pair studies and family-based segregation analyses have showed clear evidence of a heritable component in the susceptibility of OA [5]. Researches have shown the evidence that genetic factors may play a vital role in the development of OA, although little of them have been identified so far.

Long noncoding RNAs (lncRNA) have been tentatively defined as a type of RNA molecule more than 200 nucleotides in length (while microRNAs are of 20–22 nucleotides in size) and are characterized by their complexity and diversity of sequences and mechanisms of action [6]. Several lncRNAs have been reported play an important role in the pathogenesis of osteoarthritis [7, 8]. Aberrant expression profile of lncRNAs in damaged bone and cartilage of OA patients has been reported recently, indicating its potential contributions in OA development and a promising target for disease diagnosis and treatment [9]. However, the role of lncRNAs played in cartilage metabolism and their overall contributions to the degradation of chondrocyte extracellular matrix and the pathogenesis of OA are still not fully understood. Considering the important roles lncRNA played in cartilage anabolism and catabolism, we hypothesized that single nucleotide polymorphisms (SNPs) in lncRNA gene may individually or jointly contribute to the risk of osteoarthritis.

RegulomeDB, a database integrates a big collection of regulatory information from ENCODE and other data sources, is a powerful tool to choose functional SNPs in a specified chromosome region [10]. RegulomeDB presents a scoring system with categories ranging from 1 to 6 by the way of integrated annotations data on methylation, chromatin structure, protein motifs and binding. The lower RegulomeDB score indicates the stronger evidence for a variant to be located in a functional region.

Recently, many studies have used RegulomeDB to identify causal polymorphisms associated with human diseases such as cancer, polycystic ovary syndrome and so on [11, 12]. Thus in this case-control study, we explored KOA risk with lncRNA polymorphisms which are annotated in RegulomeDB.

## Methods

### Patients

In this study, Han Chinese KOA patients were recruited between June 2013 and August 2016 at the orthopaedics department, Changzhou No.1 people’s hospital according to the American College of Rheumatology (ACR) diagnostic criteria for KOA [13]. Each patient had taken the affected knee’s anteroposterior weight-bearing radiographs. Two orthopedics doctors provide a Kellgren–Lawrence (KL) score ranging from 0 to 4 assessed by the radiographic data [14]. Other knee joint diseases were excluded such as inflammatory arthritis (septic arthritis, rheumatoid, or autoimmune disease), posttraumatic arthritis, or knee joint developmental dysplasia. All healthy volunteer controls were frequency-matched to the case subjects by gender and age. The healthy control subjects reported no history of OA or other rheumatic diseases, which were included from the same hospital during the same period. Each participant was interviewed face-to-face to gather demographic data by two trained investigators. After signing informed consent at recruitment, peripheral blood of each participant was collected. All subjects were weighed with a calibrated beam balance to 0.1 kg, wearing the least possible clothes. Also their height was measured in centimeters using a stadiometer. The body mass index (BMI) was then calculated using the above data. This study was approved by the Human Research Ethics Committees of the Changzhou No.1 people’s hospital.

### LncRNAs and SNPs selection

We retrieved relevant articles from the PubMed, Medline and Embase databases up to Jul 2017 investigating the association between lncRNA and the risk of osteoarthritis. There are 15 lncRNAs reported related with osteoarthritis including UFC1, lncRNA-MSR (TMSB4XP6), PCGEM1, MEG3, HOTAIR, GAS5, PMS2L2 (uc011kep.2), RP11-445H22.4 (ENST00000427303), H19, CTD-2574D22.4 (ENST00000567795), lncRNA-CIR, HOTTIP, PACER, CILinc01 and CILinc02 [15–19].

Variant with lower RegulomeDB score indicates the variant is more strongly located in a functional region. Consequently, we selected SNPs with RegulomeDB scores ranging from 1 to 2b. Based on the data from UCSC database (GRCh37/hg19), regulomeDB database, the criteria of minor allele frequency (MAF) > 0.05 and linkage disequilibrium (LD) < 0.8 in Han Chinese, we found 10 potentially functional SNPs in these 15 lncRNAs.

### DNA extraction and genotyping

Genomic DNA was extracted from leukocyte pellets by proteinase K digestion and phenol chloroform as described in previous study [20]. The genotype detecting was performed by Sequenom MassARRAY assay without knowing the status of case or control according to instructions of the manufacturer. Three SNPs genotyping were failure due to probe design. Finally, the left 7 SNPs successfully genotyped call rate were all above 95%. Over 10% specimens were randomly slected to be repeated with over 99% consistency and two blank (water) controls were tested in each 384-well plate for quality control.

### Statistical analyses

The χ^2^ test and Student’s t-tests were used to analyze distribution differences of demographic characteristics and genotypes between cases and controls, for categorical variables and continuous variables respectively. Hardy–Weinberg equilibrium (HWE) was tested using a goodness-of-fit χ^2^ test to compare the expected and observed genotype frequencies in controls for the distribution of each SNP. We use logistic regression to estimate odd ratios (ORs) and 95% confidence intervals (CIs) to evaluate the association with KOA susceptibility after adjusted for age, sex and BMI. We use the χ^2^-based Q-test to measure the heterogeneity (ORs and 95% CIs) derived from corresponding subgroups to examine the differences between different subgroups. We also assessed the cumulative effects of the genotyped 6 SNPs using a risk score analysis with a linear of the SNP genotypes (coded as 0, 1, and 2) weighted by the regression coefficient. All of the statistical analyses were performed with SPSS Statistics Version 17.0 or PLINK (http://www.cog-genomics.org/plink2/) software.

## Results

The distribution of selected clinical variables between OA cases and controls was summarized in Table 1. In brief, the age and gender between two groups were comparable (*P* > 0.05).

**Table 1:** Distributions of select variables in OA cases and controls

Variables	Case	Control	*P*
	*n* = 278 (%)	*n* = 289 (%)	
Age, year (mean ± SD)	62.00 ± 10.55	61.13 ± 10.92	0.34
Gender			0.87
Male	82(29.5)	87(30.1)	
Female	196(70.5)	202(69.9)	
BMI	24.97 ± 3.26	23.84 ± 2.96	**< 0.01**
< 25	150(54.0)	189(66.5)	< 0.01
≥ 25	128(46.0)	95(33.5)	
KL classification			
1–2	135		
3–4	143		

Bold font presents *P* < 0.05

Rs2067079 of GAS5 was excluded due to violation of Hardy–Weinberg equilibrium. The genotype distributions of the left 6 SNPs and their associations with KOA risk were presented in Table 2. The success rates of genotyping for all these SNPs were above 98%. Logistic regression analyses revealed that H19 rs2067051 T allele was significantly associated with decreased risk of KOA adjusted for age, gender and BMI in recessive genetic model (OR = 0.63, 95% CI: 0.42–0.97, *P* = 0.03) and additive genetic model (OR = 0.79, 95% CI: 0.64–0.98, *P* = 0.03). MEG3 rs4378559 T allele was significantly associated with the increased risk of KOA in additive genetic model without adjustment (OR = 1.32, 95% CI: 1.01–1.74, *P* = 0.04), but the association no longer existed after adjustment (*P* = 0.08). HOTTIP rs2023843 C allele showed boundary positive in additive genetic model after adjustment (OR = 1.23, 95% CI: 0.96–1.58, *P* = 0.10).

**Table 2:** Logistic regression analysis of associations between selected polymorphisms and KOA risk

SNP	Genotype	Case	Control	OR (95%CI)	*P*	OR (95%CI)^a^	*P* ^a^
H19 rs2067051	CC	162	142	1		1	
	TC	64	63	0.89(0.59–1.35)	0.58	0.87(0.57–1.35)	0.54
	TT	50	69	0.64(0.41–0.97)	**0.04**	0.61(0.39–0.95)	**0.03**
	TT + TC vs.CC			0.76(0.54–1.06)	0.11	0.73(0.52–1.04)	0.08
	TTvs.TC+ CC			0.66(0.44–0.99)	**0.05**	0.63(0.42–0.97)	**0.03**
	Additive			0.81(0.66–0.99)	**0.04**	0.79(0.64–0.98)	**0.03**
MEG3 rs4378559	CC	148	176	1		1	
	TC	108	98	1.31(0.92–1.86)	0.13	1.23(0.86–1.76)	0.26
	TT	21	14	1.78(0.88–3.63)	0.11	1.78(0.86–3.68)	0.12
	TT + TC vs.CC			1.37(0.98–1.91)	0.07	1.30(0.92–1.83)	0.13
	TT vs.TC+ CC			1.61(0.80–3.23)	0.18	1.65(0.81–3.35)	0.17
	Additive			1.32(1.01–1.74)	**0.04**	1.28(0.97–1.69)	0.08
MEG3 rs4906024	CC	88	80	1		1	
	TC	103	131	0.71(0.48–1.06)	0.10	0.72(0.48–1.09)	0.12
	TT	82	76	0.98(0.64–1.52)	0.93	1.04(0.67–1.62)	0.87
	TT + TC vs.CC			0.81(0.57–1.17)	0.26	0.84(0.58–1.21)	0.35
	TT vs.TC+ CC			1.19(0.82–1.72)	0.35	1.25(0.86–1.83)	0.24
	Additive			0.99(0.79–1.23)	0.90	1.02(0.81–1.27)	0.90
HOTTIP rs10233387	AA	62	80	1		1	
	GA	127	137	1.20(0.79–1.80)	0.39	1.22(0.80–1.85)	0.35
	GG	64	62	1.33(0.82–2.16)	0.24	1.49(0.91–2.45)	0.11
	GG + GA vs.AA			1.24(0.84–1.82)	0.28	1.30(0.88–1.93)	0.19
	GG vs.GA+ AA			1.19(0.79–1.77)	0.41	1.31(0.87–1.98)	0.20
	Additive			1.16(0.91–1.47)	0.24	1.22(0.95–1.57)	0.11
HOTTIP rs2023843	TT	92	108	1		1	
	CT	143	144	1.17(0.81–1.67)	0.41	1.23(0.85–1.78)	0.28
	CC	43	37	1.36(0.81–2.30)	0.24	1.52(0.89–2.58)	0.13
	CC + TC vs.TT			1.21(0.85–1.70)	0.29	1.29(0.90–1.83)	0.17
	CC vs.TC+ TT			1.25(0.78–2.00)	0.36	1.34(0.83–2.18)	0.24
	Additive			1.17(0.91–1.49)	0.22	1.23(0.96–1.58)	0.10
HOTAIR rs10783618	CC	157	159	1		1	
	TC	96	108	0.90(0.63–1.28)	0.56	0.90(0.63–1.29)	0.56
	TT	25	22	1.15(0.62–2.13)	0.65	1.18(0.63–2.20)	0.61
	TT + TC vs.CC			0.94(0.68–1.31)	0.73	0.94(0.67–1.33)	0.74
	TT vs.TC+ CC			1.20(0.66–2.18)	0.55	1.23(0.67–2.26)	0.51
	Additive			1.00(0.77–1.29)	0.99	1.00(0.77–1.30)	0.98

Bold font presents *P* < 0.05

^a^Adjusted for age, gender and BMI

Further stratified analyses were conducted in four positive models as above mentioned (Table 3). Results showed that H19 rs2067051 T allele on KOA was more evidenced for female in recessive model (TT vs.TC+ CC) (*P* for heterogeneity was 0.01). MEG3 rs4378559 T allele on KOA risk was more obvious in BMI ≥ 25 in additive model (*P* for heterogeneity was 0.02). HOTTIP rs2023843 C allele on KOA was more distinct for younger age (age < 60) in additive model (*P* for heterogeneity was 0.03).

**Table 3:** Stratified analyses of rs2023843, rs2067051, and rs4378559 genotypes associated with patients of kOA by selected variables

	rs2067051 Additive			rs2067051 TTvs.TC+ CC			rs4378559 Additive			rs2023843 Additive		
	OR(95%CI)^a^	*P* ^a^	*P*_het_^b^	OR(95%CI)^a^	*P* ^a^	*P*_het_^b^	OR(95%CI)^a^	*P* ^a^	*P*_het_^b^	OR(95%CI)^a^	*P* ^a^	*P*_het_^b^
Age			0.12			0.18			0.17			**0.03**
age < 60	0.63(0.45–0.89)	0.01		0.41(0.21–0.84)	0.01		1.04(0.68–1.61)	0.86		1.64(1.11–2.44)	0.01	
age ≥ 60	0.90(0.68–1.19)	0.47		0.77(0.44–1.33)	0.34		1.56(1.07–2.27)	0.02		0.92(0.65–1.30)	0.63	
Gender			0.12			**0.01**			0.76			0.07
male	1.09(0.70–1.71)	0.69		1.92(0.75–4.91)	0.18		1.36(0.80–2.31)	0.25		1.78(1.09–2.90)	0.02	
female	0.73(0.57–0.93)	0.01		0.48(0.29–0.78)	0.00		1.24(0.89–1.72)	0.20		1.05(0.78–1.42)	0.75	
BMI			0.43			0.13			**0.02**			0.55
BMI < 25	0.76(0.57–1.00)	0.05		0.49(0.28–0.87)	0.01		1.02(0.72–1.43)	0.92		1.15(0.84–1.59)	0.38	
BMI ≥ 25	0.90(0.64–1.26)	0.54		0.96(0.50–1.86)	0.91		2.07(1.27–3.38)	0.00		1.35(0.90–2.04)	0.15	
KL			0.20			0.50			0.17			0.22
KL12	0.70(0.53–0.92)	0.01		0.56(0.32–0.98)	0.04		1.07(0.75–1.52)	0.72		1.41(1.03–1.93)	0.03	
KL34	0.89(0.69–1.16)	0.40		0.73(0.44–1.22)	0.23		1.51(1.07–2.11)	0.02		1.07(0.78–1.46)	0.68	

Bold font presents *P* < 0.05

^a^Adjusted for age, gender and BMI

^b^*P* value for heterogeneity test

Cumulative effects of the 6 SNPs were conducted using a risk score analysis weighted by the regression coefficient (Table 4). The mean of cumulative risk score among KOA cases (0.38 ± 0.40) was higher than that among controls (0.29 ± 0.34) (*P* value for T test < 0.01). Logistic regression analyses revealed that risk score was significantly associated with increased risk of KOA adjusted for age, gender and BMI (OR = 2.21, 95% CI: 1.36–3.60, *P* < 0.01).

**Table 4:** Cumulative risk scores of 6 SNPs on the risk of kOA

	Case	Control	*P* ^a^	OR(95%CI)^b^	*P* ^b^
Risk score	0.38 ± 0.40	0.29 ± 0.34	**< 0.01**	2.21(1.36–3.60)	**< 0.01**

Bold font presents *P* < 0.05

^a^*P* for T test

^b^Adjust for age, gender, and BMI

## Discussion

In the current study, we conducted a case-control study to explore the association between genetic variants identified by RegulomeDB in candidate lncRNA genes and KOA. Our results confirm that putative functional variants H19 rs2067051 and MEG3 rs4378559 were associated with KOA susceptibility. Heterogeneity existed between different gender, BMI and age group for H19 rs2067051, MEG3 rs4378559 and HOTTIP rs202384 respectively, which means gene-environment interactions between genetic variants in lncRNA genes and these clinical data for KOA risk.

All RegulomeDB score of H19 rs2067051, MEG3 rs4378559 and HOTTIP rs2023843 were 2b. ChIP-seq data showed these polymorphisms binding to many proteins including EZH2, E2F6, REST and IKZF1 (http://regulome.stanford.edu/).

The development of osteoarthritis was ameliorated by inhibition of EZH2 through the Wnt/β-catenin pathway [21, 22]. E2F6 encodes a member of transcription factors which play an important role in the cell cycle controling [23]. A transcriptional repressor which represses neuronal genes in non-neuronal tissues was encoded by REST [24]. IKZF1 encodes a transcription factor which belongs to the zinc-finger DNA-binding protein family, which correlates with chromatin remodeling [25]. IKZF1 participated in inflammation suggested that IKZF1 may contribute to osteoarthritis [26].

A 2.5 kb RNA Polymerase II dependent transcript was expressed from H19 and it is spliced, capped, polyadenylated, and finally exported into the cytoplasm [27]. MiR-675, which suppresses growth, is developmentally reversed in H19 [28]. It also participates in the development of OA through affecting the pathological processes including inflammatory response, extracellular matrix (ECM) disruption, angiogenesis and apoptosis. Research showed that H19 was up-regulated in OA compared with normal tissue and was a metabolic marker in damaged cartilage of OA patients or in cultured chondrocytes under hypoxic signaling condition [29, 30]. Rs2067051 of H19 has been verified connected with birth weight and coronary artery disease [31, 32]. Both high birth weight and coronary artery disease have relationship with higher BMI, which are crucial risk factors for KOA. In this study, we also found heterogeneity was existed for H19 rs2067051 in different gender group. It is already found that H19 represents a key factor which causes sex differences in the incidence of cholestatic liver injury in mice whose multidrug resistance 2 gene was knockout [33].

A maternally expressed lncRNA, which was named MEG3, was closely connected to inflammation-related diseases, for example knee osteoarthritis [34]. It revealed that MEG3 demonstrated low expression level in OA cartilage samples in rat model. It was also shown that MEG3 was down regulated and negatively correlated with VEGF expression levels in cartilage samples from knee osteoarthritis patients [34]. MEG3 knockdown could promoted chondrocytes proliferation and inhibited chondrocytes apoptosis induced by IL-1β possibly through miR-16/SMAD7 system [35].

HOTTIP, which participate in the process of osteoarthritis advance and endochondral ossification, showed higher expression level in OA cartilage in comparison to normal cartilage [36]. Through modulating intergrin-α1 either transcriptionally by HOXA13 or epigenetically by DNMT-3B, HOTTIP control cartilage destruction and development. Research data also indicated that HOTTIP could be potential diagnostic biomarker or therapeutic target for OA and other joint cartilage related disease.

## Conclusions

In conclusion, our study suggested that potential functional genetic variation in lncRNA plays a crucial role in the pathogenesis of KOA. Further independent studies with different races and larger sample sizes are necessary to elucidate the molecular mechanisms underlying these findings.

## Abbreviations

BMI: Body mass index
CIs: Confidence intervals
ECM: Extracellular matrix
HWE: Hardy–Weinberg equilibrium
KOA: Knee osteoarthritis
lncRNA: Long noncoding RNAs
ORs: Odd ratios
SNP: Single nucleotide polymorphisms
